# A randomized study of digital versus genetic counselor return of actionable genetic research results to biobank participants (RESPECT3 study)

**DOI:** 10.1186/s12910-026-01439-x

**Published:** 2026-03-31

**Authors:** Anuja Rajendra Godbole, Elisabeth Wood, Brian Egleston, Lily Hoffman-Andrews, Sarah Brown, Sarah Howe, Sanjana Shastri, Rajia Mim, Justin Feng, Anjali Owens, Susan Domchek, Reed Pyeritz, Bryson W. Katona, Staci Kallish, Giorgio Sirugo, JoEllen Weaver, Linda Fleisher, Kuang-Yi Wen, Elena Elkin, Katherine L. Nathanson, Daniel J. Rader, Angela R. Bradbury

**Affiliations:** 1 Abramson Cancer Center and Division of Hematology-Oncology, University of Pennsylvania, Philadelphia, PA USA; 2 Fox Chase Cancer Center, Temple University, Philadelphia, PA USA; 3 Division of Cardiovascular Medicine, University of Pennsylvania, Philadelphia, PA USA; 4 Division of Translational Medicine and Human Genetics, University of Pennsylvania, Philadelphia, PA USA; 5 Division of Gastroenterology, University of Pennsylvania, Philadelphia, PA USA; 6 Thomas Jefferson University, Philadelphia, PA USA; 7 Columbia University, New York, NY USA; 8 Department of Medical Ethics and Health Policy, University of Pennsylvania, Philadelphia, USA

**Keywords:** Genetic research results, Return of results, Digital intervention, Institutional biobank, Chatbot

## Abstract

**Background:**

There is consensus that research participants should be informed about plans for return of genetic research results. However, best practices for return of results in large biobank and cohort studies do not exist currently, and how best to communicate actionable genetic research results remains unclear. While having genetic counselors disclose these results may be ideal to ensure understanding, minimize distress, and optimize medical follow-up, genetic counselor (GC) workforce shortages and costs are barriers. The RESPECT3 study evaluates whether digital delivery alternatives for pre-disclosure education and return of actionable genetic research results is non-inferior to remote telehealth disclosure by a genetic counselor.

**Methods:**

The RESPECT3 Study is a hybrid type 1 effectiveness-implementation study which evaluates digital alternatives for pre-disclosure education and return of actionable research results in randomized non-inferiority trial. The return of results process in RESPECT3 uses a two-step process. In step one, participants with an actionable result and procedural controls (no actionable result) are invited to digital pre-disclosure education and provided options for opting out of results. In Step 2, those with actionable results who have not opted out are randomized to receive results via a digital disclosure intervention (digital-ROR) or with a GC. Participants include English-speaking adults who enrolled in the Penn Medicine BioBank (PMBB) and have an actionable research result according to the ACMG secondary findings list. The primary outcomes are non-inferiority of knowledge and disease specific distress (cancer and cardiovascular disease) after receipt of research results. Uptake of research results is a secondary non-inferiority outcome. Confirmatory clinical testing is covered by the study and all participants with confirmed results are referred to the appropriate clinical genetic program for clinical recommendations.

**Discussion:**

The RESPECT3 study is expected to provide critical empiric data on the effectiveness of using digital alternatives for pre-disclosure education and return of genetic research results in a representative clinical population of research participants who enrolled in an institutional biobank where return of results was not the focus of the study. Equally important, our theoretically-informed process evaluation data is expected to inform modifications to identify barriers and facilitators to broader implementation.

**Trial registration:**

NCT04242667.

**Supplementary Information:**

The online version contains supplementary material available at 10.1186/s12910-026-01439-x.

## Background

Large prospective cohort studies that have banked DNA are using advance genetic sequencing to better understand the effects and interplay of genes, the environment and lifestyle on health outcomes [1, 2]. The hope for personalized medicine is to tailor prevention, treatment or screening recommendations for individual patients based on their genotype or other genomic profiles, which will ultimately result in modified health behaviors (e.g. risk reducing behaviors or screening) and improved patient outcomes [3–5]. However, genetic sequencing can identify actionable genetic variants that are likely to have medical impact on the research participants and their families.

The potential to incidentally identify “actionable” genetic variants (i.e. those that are likely to have a medical impact and are amenable to medical intervention or screening) has raised questions regarding obligations to share such findings with research participants and how it should be shared [2, 6–11]. Several studies suggest that many research participants are interested in receiving at least some genetic research results [12–19]. Based on principles of autonomy and respect for persons [14, 15, 20–26], there is consensus that research participants should be informed about the return of genetic research results, including the type of results, the process, and the option to decline [6, 8, 27–34]. However, best practices for implementation of these recommendations in large biobank and cohort studies do not exist currently. Most programs send participants a letter outlining a plan to return results and the option to “opt-out” [35–37], which may not suffice for well-informed decision making, particularly when details at consent were limited and the ACMG actionable results list covers a range of cancers, cardiovascular and other medical conditions [35, 38–41]. Pre-test counseling by genetic counselor (GCs) is a standard that could be applied to ensure informed decision making (e.g. advantages). However, this approach is costly and impractical for research programs and alternative cost-effective strategies are needed [42]. 

Similarly, how best to communicate genetic research results to research participants remains unclear. Unlike patients with clinical genetic testing, research participants may not be prepared for actionable results, particularly if the results are unrelated to the reason they enrolled in research, or if they enrolled as healthy unselected participants many years prior (e.g. large biobanks). While having GCs disclose these results may be ideal to ensure understanding, minimize psychosocial distress, and optimize medical follow-up and cascade testing [43], GC workforce shortages and costs are barriers [43, 44]. Many programs have used GCs to share genetic research results [8, 35, 37, 45–47], while others have provide results to local health care providers [48, 49] who may lack the necessary expertise or resources, resulting in suboptimal patient outcomes [50]. Interactive patient-centered digital interventions and/or videos developed by genetic providers may provide a cost-effective alternative [51–54]. Further, these interventions could be paired with genetic counseling for participants with remaining questions, while still reducing overall provider time. Potential disadvantages include poor understanding, increased test-related short-term and/or long-term affective responses or suboptimal post-test behaviors (e.g. medical management and cascade testing). Limited data exist on patient outcomes, clinical outcomes, and costs associated with various approaches. Empirical data are needed to inform best approaches for pre-disclosure education and returning results to realize benefits for participants.

Although research participants have reported high interest in receiving research results [15, 48, 55–62], uptake has been lower when results have been offered (48–86%), particularly in biobank studies where return of results was not emphasized during enrollment and uptake has generally been around 50% [36, 38, 63–69]. There are limited patient-reported outcomes in research settings [63–65, 70], although some genomic implementation studies report no psychological harms with return of genetic findings [71, 72]. In the RESPECT2 study, research participants could choose pre-disclosure education with a GC or a patient-centered digital intervention. All participants received results with a GC. Favorable cognitive (e.g. knowledge) and affective (e.g. distress and uncertainty) responses were found, although the number of actionable results was small [73, 74]. 

### Objectives

The goal of the RESPECT3 Study is to conduct a Hybrid Type 1 effectiveness-implementation study to evaluate digital delivery alternatives for pre-disclosure education and return of actionable genetic research results. Based on our preliminary data and related research, we include both digital pre-disclosure education interventions and a private web-based portal for return of individual research results as an alternative to result disclosure by a genetic provider. Additionally, the study incorporates three chatbots designed to increase completion of pre-disclosure education, receipt of results and clinical confirmation testing.

#### Specific aim 1

Our primary aim is to evaluate in a randomized study whether disclosure of actionable genetic results by a digital intervention (digital-ROR) provides non-inferior short-term and longitudinal outcomes (knowledge, psychological distress, health and psychosocial behaviors and costs) compared to remote telehealth disclosure by a genetic counselor (GC)(e.g. usual care).

#### Specific aim 2

Our secondary aims are to evaluate the uptake of supplemental digital education (offered through a digital intervention or a chatbot) among Penn Biobank research participants notified of the option to opt-out of receipt of actionable genetic research results (Aim 2a), to evaluate the frequency of opting-out of receipt of actionable genetic research results (Aim 2b) and to evaluate the impact of use of digital education on opting out of receipt of actionable results (Aim 2c).

#### Specific aim 3

Our third aim is to conduct a multi-stakeholder mixed-methods process evaluation to understand the potential moderators (e.g. intervention usage, sociodemographic factors, genetic test result) of short-term and longitudinal outcomes to understand who benefits more or less from digital education and digital return of results (Aim 3a), and to understand the facilitators and barriers to implementation of digital interventions for return of actionable genetic research results and recommendations for future adaptation and sustainability (Aim 3b).

## Methods

### Study design

**Fig. 1:**
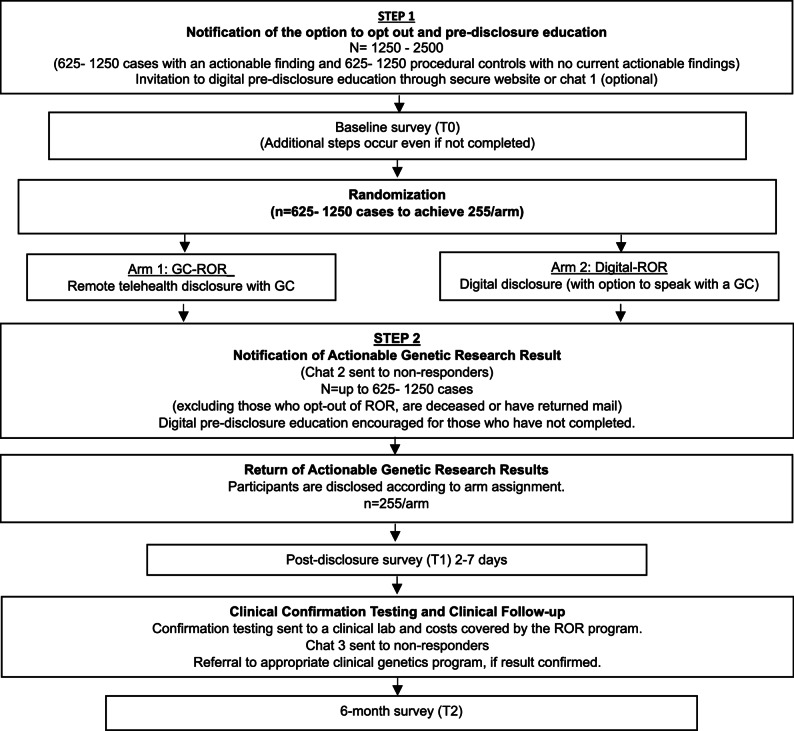
Schema

The RESPECT3 Study is a Hybrid Type 1 effectiveness-implementation study, to evaluate digital alternatives for pre-disclosure education and return of actionable research results in a prospective randomized trial. The study includes a concurrent CFIR (Consolidated Framework for Implementation Research)-informed process evaluation to consider future implementation and sustainability of using eHealth interventions for return of actionable genetic research results in large and sociodemographically diverse research cohorts. We use a two-step process to notify biobank participants of the return of results process. In Step 1, participants with an actionable result and procedural controls (no actionable result) are invited to digital pre-disclosure education and provided options for opting out of results. In Step 2, those with actionable results who have not opted out are randomized to receive results via a digital disclosure intervention (digital-ROR) or with a GC (Fig. 1).

### Ethics approval

The original PMBB consent stipulated that actionable results would be returned but did not describe the various types of results or when and how these would be returned. It also did not specify how one might decline (e.g. opt out of) results. Thus, the Return of Results (ROR) protocol is an extension of the original PMBB protocol and consent process. It is not “a new study” but rather is a process for implementing the obligation that was already established in the PMBB consent, when it was stated that results that could impact one’s health would be returned. Thus, the ROR process provides PMBB participants with current and comprehensive information on what results are being returned and how and the opportunity to decline these results if they desire. These activities (with the exception of the surveys) are all a direct extension of the obligation set in the original consent regarding results. We do require an informed consent for the surveys, but if a participant declines the surveys, we are still bound by the original PMBB consent to provide actionable results as previously stated. The participants will receive a $50 gift card for each survey completed.

### Digital pre-disclosure education and return of results intervention

**Fig. 2:**
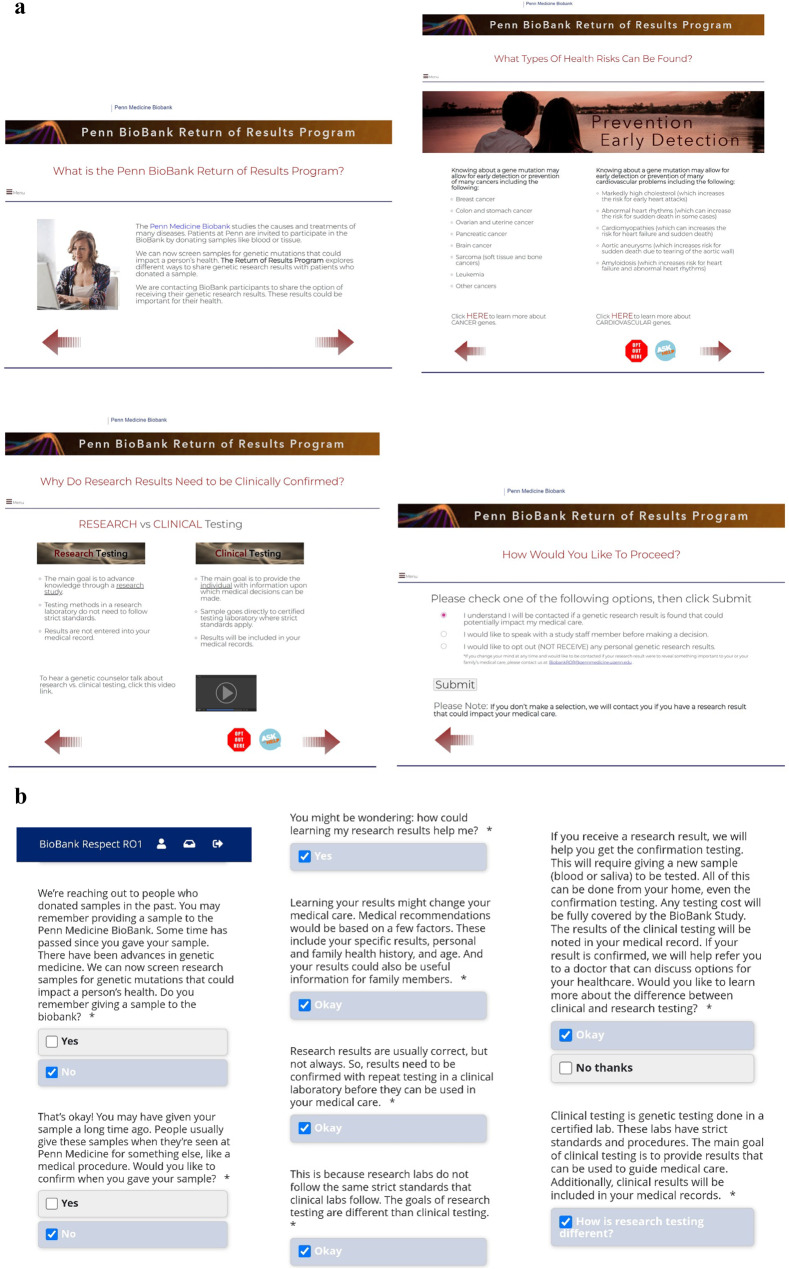
Digital pre-disclosure education intervention. **a** Web pre-disclosure education intervention. **b** Chatbot (Chat 1) pre-disclosure education intervention

**Fig. 3:**
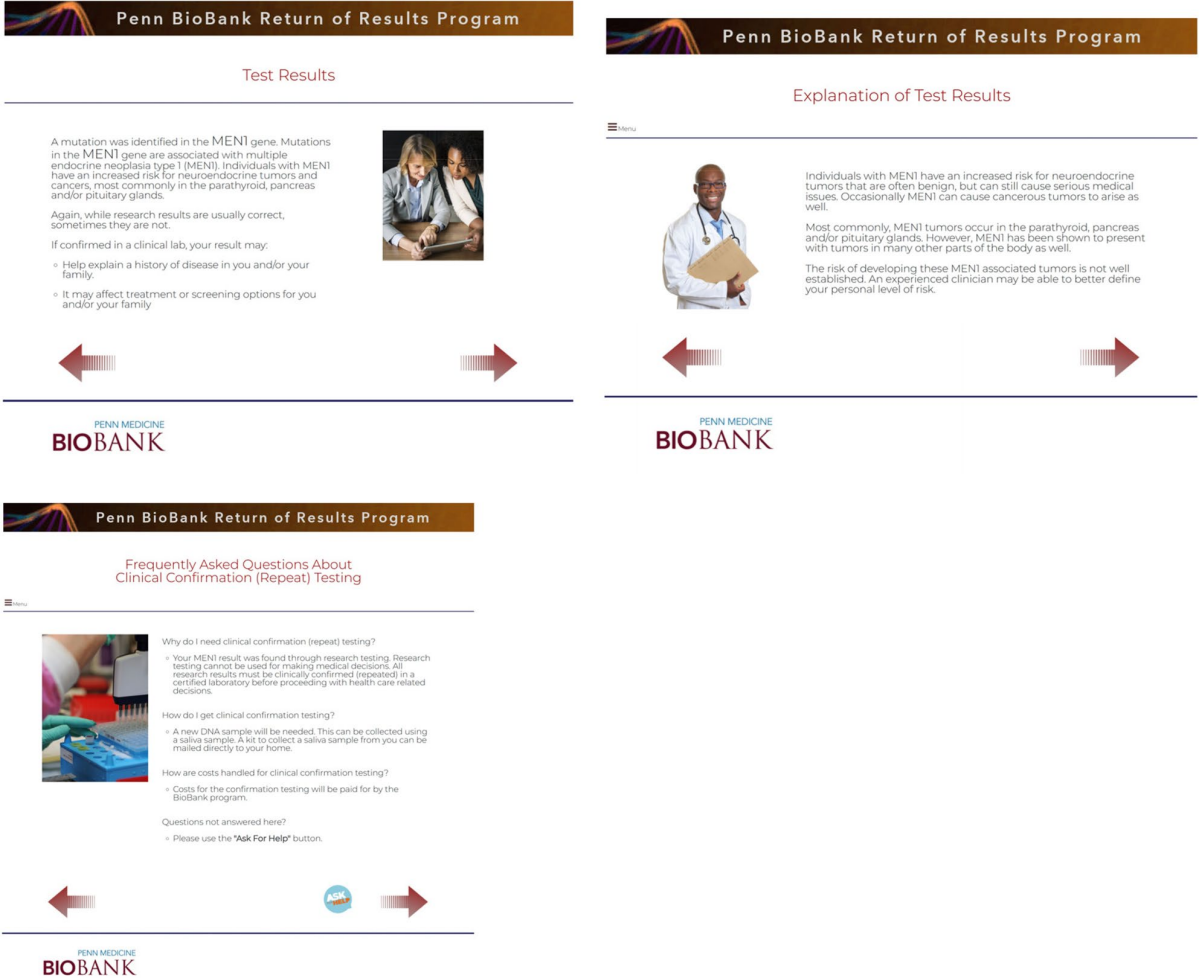
Digital return of results (ROR) intervention

Informed by prior research, we developed a mobile-ready, patient-centered multi-modality digital pre-disclosure education and return of results intervention (digital-ROR) as alternatives to genetic counseling to reduce participant burdens and steps to receiving actionable genetic research results. The study team adapted the digital education tool for this study from the stakeholder-informed RESPECT multi-modality pre-disclosure web education (e.g., digital) intervention and modified it based on user and usability testing with biobank participants. Digital-ROR was specifically developed for this study. Both digital interventions cover the same content as genetic counseling and were developed with patient feedback to meet the needs of a diverse clinical population. Informed by the tiered-binned model, the interventions include ‘‘indispensable’’ Tier 1 information presented to all participants, and ‘‘optional’’ Tier 2 information for varying needs. The digital pre-disclosure education intervention consists of six modules and nine optional videos (Table 1; Figs. 2a-b). The digital pre-disclosure education reviews in more detail the types of actionable results being returned, the differences between research and clinical testing, the need for confirmation testing and the benefits, risks and limitations of receiving actionable genetic research results and study steps and procedures. The digital-ROR intervention has four modules and three optional videos (Table 2; Fig. 3). It shares the actionable mutation identified, associated risks and general management approaches. It also emphasizes the need for clinical confirmation testing and medical follow-up. To help ensure access, the digital interventions are optimized for use by mobile devices and various web browsers and are accessible through an individual user ID and passcode. The digital-ROR intervention has an additional birth month and year field which serves as an added layer of privacy. To help ensure accuracy and quality, all actionable research results are entered into a case report form and uploaded for each randomized participant by a study staff member and a second study staff member confirms accuracy of the actionable genetic research result and uploaded research result in the study participant’s private web-portal. At least one of these staff members is a genetic counselor and these steps occur for each result uploaded into the private web-portal prior to the result being accessible by the study participant.

**Table 1:** Digital pre-disclosure education intervention (website)

Module	Tier 1 Content (# screens)*	Tier 2 Content (# screens)	Tier 2 Videos**[minutes]
WELCOME/LANDING PAGE	NA	NA	NA
1: Introduction	-What is the Penn Biobank Return of Results Program? (2) -What to expect (overview of content and option to opt out) (1) -Option to speak with a person (1)		
2: Genetics Overview	-Genes and inheritance (7) -Genes and health risks (1)		-Basic genetics overview [1:19]
3: Types of results and health implications	-What types of genes and conditions could be found (1) -How can genes impact health? (1) -How might learning my research results help me? (1)	-More information on cancer genes (1) -More information on cardiovascular genes (1)	-How cancer genes affect health [1:46] -How cardiovascular genes affect health [1:08]
4: Research v. clinical testing	-Need for confirmation testing (1) -Research v. clinical testing (1) -Medical follow-up (1) -How costs of confirmation testing will be covered (1)	-More information on medical care options for cancer genes (1) - More information on medical care options for cardiovascular genes (1)	-Research v. clinical testing [1:52] -Medical care for cancer genes [0:51] -Medical care for cardiovascular genes [0:56]
5: Risks, benefits and limitations of receiving results	-Potential benefits of receiving results (1) -Potential limitations of receiving results (1) -Potential risks of receiving results (1)		-Benefits [0:16], limitations [0:46] and risks [0:36] of receiving results
6: Next steps	-Overview: What’s next? (1) -What happens after I receive my results? (2) -HIPAA/privacy (3) -How would you like to proceed? ^ (1) -Survey request (1)		
Optional Content		Glossary of terms	

The intervention is informed by the tiered-binned model for genetic education and informed consent and was reviewed with cancer genetics providers and experts in health disparities and health communication. Readability testing was completed for all content and modifications made to achieve a readability score of 9th grade or lower. The linear digital intervention includes six modules and optional videos. Participants can view modules for as long, and as many times as desired. They can also go back to previously viewed topics

*All pages offer “Ask for Help” option

**Videos include a genetic counselor explaining specific topics. Content in some videos are intentionally redundant to Tier 1 and Tier 2 content and designed to provide an alternative method for reviewing content for participants with different learning preferences

^Participants can choose to: (a) opt out of receiving results, (b) speak with a staff member/GC to make a decision, (c) acknowledge they have enough information and understand they will be contacted if something is found in their sample

Completing Tier 1 content takes 8–14 min; completing Tier 1 and 2 content (excluding videos) takes 11–17 min

**Table 2:** Digital return of results (ROR) intervention

Module	Tier 1 Content (# screens)*	Tier 2 Content (# screens)
1: Welcome Page/ Instructions	-Overview and option to speak with a genetic counselor (1) -How to use this website (1) -Option to review education^ (2) -Option to review specific education items (1)	-Basic genetics^^ -How genes impact health^^ -Research v. clinical testing^^ -Genetic mutations and medical care^^ -Benefits [0:16], limitations [0:46] and risks [0:36]^^
2: Test Result	-Ready to receive your results? (option for more time or to speak with a GC) (1) -Genetic test result (1)	-Copy of test report
3: Explanation of Result:	- Implications if result is confirmed (2–3 screens based on gene^#^) -What does this mean for my relatives? (1)	-Risks and guidelines
4: Next Steps	-Need for confirmation testing and medical follow-up if confirmed (1) -FAQ for confirmation testing (1) -Are you ready for confirmation testing? (yes, send me a kit or no, I would like to speak with a person)	-PDF of clinical test result (confirmation testing) available for download once result is available

*All pages offer “Ask for Help” option

^If participants have not previously viewed the digital pre-disclosure education intervention they have to actively decline education on this screen. If they decline, they are provided a single screen with key points (minimum information)

^^refers to duplicate screens from education website

^#^Option to select Risks and Guidelines for females v. males for some genes

Completing Tier 1 content takes 1–5 min; completing Tier 1 and 2 content (excluding videos) takes 5–12 min

Three chatbots, delivered by SMS, are designed to increase completion of pre-disclosure digital education, receipt of results and confirmation testing (Table 3). The education chat (Chat 1) includes the same information as the digital pre-disclosure education intervention but accessible via an SMS text to provide a different modality for accessing this information. The results reminder chat (Chat 2) includes instructions for receipt of results via an SMS text, and the confirmation testing follow-up chat (Chat 3) includes information on confirmation testing along with an option to view the commonly asked questions accessible via an SMS text.

**Table 3:** Chatbot

Chat	Purpose	Content Summary*	Reminders
1: Chat 1 (Education chat)	Chat 1 (education chat) offers a summary of BioBank’s plan to return research results if available and next steps, while also providing an option to opt out or request assistance from study staff.	Participants are provided with an external link to an interactive form which has similar education content represented in Table 1.	-Requires Self-enrollment to Way to Health platform by participant. -There are no reminders sent if the chat is incomplete.
2: Chat 2 (Results reminder chat)	Chat 2 reminds participants of communication being sent about available research results.	- Informs that there is a research result available to learn (1) -Confirm if participant has received study communication (yes or no) (1) -Offer to resend the communication by email or mail (2) -Importance of confirmation testing and process (2) -Study team contact information (1)	-Includes initial notification 7 days after letter 2 is sent, followed by three reminders on days 5, 23, and 53.
3: Chat 3 (Confirmation Testing Follow-Up)	Chat 3 follows up with participants proceeding forward with confirmation testing.	-Confirm if participant received clinical confirmation testing kit (yes or no) (1) -Offer to help if participant has not received the testing kit (2) -Confirm if participant has mailed clinical confirmation testing kit back to laboratory (1) -Importance of clinical confirmation testing and commonly asked questions (2) - Study team contact information and confirm that participant is ready to send kit back if they have not already (1) -Ask reason for not proceeding forward with clinical confirmation testing (1)	-Initial notification sent 7 days after confirmation testing kit is ordered, followed by 3 reminders on days 5, 23, and 53.

*All three chats are initiated with a text message that asks participants to confirm their identity and the option to switch off text messaging by replying “BYE” or asking for help from the research team at any point by replying “ASSIST”

The chatbots were initially developed and hosted through a HIPAA compliant external vendor (Invitae Gia Clinic Hub), but transitioned to an internal platform (Way to Health) in 2024

### Setting

Participants are English-speaking adults who enrolled in the Penn Medicine BioBank (PMBB), which links genomic data to electronic health record (EHR) phenotype data at the University of Pennsylvania. We reviewed whole exome sequencing (WES) for actionable variants in genes on ACMG SF v.3.0 - v3.3 [75–78]. Variants were eligible for return based on ClinVar germline classifications (pathogenic, likely pathogenic, or conflicting classifications that included ≥ 2 laboratory submissions of pathogenic/likely pathogenic) or truncating variants (PTVs) (nonsense, frameshift), excluding the last exon and the last 50 base pairs (bp) of the penultimate exon, in genes where PTVs are a disease mechanism. For autosomal recessive conditions, variants were reviewed for either homozygosity of a single variant, or two different variants both meeting eligibility for return. Selected variants were deemed ineligible if sequencing read depth was < 25, allele depth was < 5, or homozygosity for the reference allele.

### Study participants

#### Eligibility

Eligibility criteria include individuals who participated in Penn Medicine Biobank, who are 18 years or older, able to understand and communicate in English, have an actionable genetic mutation (“cases”) or have been selected as a procedural control participant, and who agreed to be re-contacted in the future or were not provided the opportunity to indicate a preference. Procedural controls allow participants to learn about the return of results program and opt out before notification of an actionable result. This step was felt to be particularly important for this study as the return of results is not a primary focus at the time of consent to the PMBB, and the types of results being returned and procedures for return have not been included at the time of the original PMBB consent.

Exclusion criteria include individuals who are noted to be deceased in the EHR, are identified as deceased after contact or who have withdrawn consent to the PMBB. We exclude subjects for whom there was evidence in the clinical record of already having received the same actionable result through routine care (e.g. clinical genetic testing).

#### Enrollment goals

Our enrollment goal for the primary non-inferiority aim (Aim 1) is 255 participants per arm. To achieve this goal, up to 2500 PMBB participants (cases and procedural controls) are expected to be contacted.

### Study steps (Fig. 1)

#### Notification of the option to decline ROR and pre-disclosure education

Eligible cases and procedural controls are sent the Step 1 Letter (e.g. opt-out notification letter) (Supplemental Fig. 1) by MyPennMedicine portal (MPM), encrypted email or mail. The letter advises potential participants of (a) the potential for researchers to identify actionable genetic research results, (b) that researchers will be returning these actionable results to individuals if such is identified in their research sample, (c) that they can elect to NOT receive these actionable results (by calling a number or via the digital intervention), and (d) that all participants will be provided the opportunity to speak with a genetic counselor to receive their results. The letter also includes a link to view the pre-disclosure digital education, and a link to education chat (Chat 1) along with a QR code directing to the same link for Chat 1. Letter 1 also includes a one-page information sheet describing key points of the study and a one-page educational FAQ about the Biobank ROR program (Supplemental Fig. 1).

Those who request to speak with a genetic counselor as an alternative (or in addition) to completing pre-disclosure digital education are scheduled for remote telehealth counseling in the home by either phone or videoconference.

#### Randomization

After at least 4 weeks, participants with an actionable finding (cases) who have not chosen to opt-out of receiving their results are randomized to receive their results by remote telehealth disclosure with a GC (Arm 1) or by a private ROR digital intervention (Digital-ROR) with the option to schedule a call with a GC at any time in the process (Arm 2). A permuted block design is used for randomization, stratifying by cancer or cardiovascular genes and sex.

Randomized participants are sent the Step 2 Letter by MyPennMedicine portal (MPM), encrypted email or mail informing them that an actionable genetic finding that could potentially impact their health has been identified (Supplemental Fig. 2). The letter includes how they will receive their actionable results (according to their randomization), the next steps for receiving results and that they can still elect to NOT receive results. Procedural controls do not receive the Step 2 Letter, but any who opt-out of results after the Step 1 Letter has their decision recorded in case they are found to have an actionable result with additional sequencing or analysis.

#### Disclosure of genetic research results

##### Arm 1 (remote telehealth disclosure with a genetic counselor)

Participants randomized to remote telehealth disclosure with a GC (Arm 1) are provided with a telephone number and email for contacting the research team to schedule their disclosure session. Appointments are offered within 1–2 days of contact, and participants can choose telephone or videoconference for their disclosure session. Participants who have not completed the pre-disclosure education (digital or chatbot) are directed to complete it, or decline it, prior to being scheduled for result disclosure with a GC. Genetic counselors use standardized pre-disclosure counseling checklists adapted from the RESPECT single-site study and utilized in our pilot study [79]. A printed genetic research result report is mailed or emailed (encrypted) to a verified email account to Arm 1 participants following completion of remote disclosure (Supplemental Fig. 3).

##### Arm 2 (digital-ROR)

Participants randomized to the digital return of results arm are provided access information to the digital intervention in the Step 2 Letter. Participants who have not completed pre-disclosure education (digital or chatbot) are directed to complete it, or decline it, prior to proceeding to the results page on the digital-ROR intervention. Participants are informed in the Step 2 Letter, that they can choose to schedule a disclosure session with a genetic counselor if preferred. They can also request a remote telehealth visit with a genetic counselor at any time (e.g. before or after receipt of results). Arm 2 participants will have the ability to print a results report directly from the digital-ROR intervention.

##### Non-response procedures

For both arms, multiple follow-up attempts are made by phone, verified email, letter, or Chat 2 to all participants who do not call to schedule their remote disclosure session or log on to digital-ROR to receive their results. The results reminder chat (Chat 2) includes an initial notification 7 days after Step 2 letter is sent, followed by three reminders on days 5, 23, and 53. Chat 2 includes ways the research team can resend the Step 2 letter to participants if they indicate their desire to receive it again by email or through mail. The research team also makes three phone call attempts to contact the participant. Participants who have exceeded these contacts and have not received their results or communicated their decision to opt-out of receiving results are sent a Closing Letter (Supplemental Fig. 4), reiterating the option to receive actionable results but that the study team will not continue to attempt to contact the participant. Contact information is provided and they are informed they can reach out to the study team in the future if they are interested in receiving their results.

#### Clinical confirmation testing

Participants who receive actionable research results are instructed that confirmatory testing in a Clinical Laboratory Improvement Amendments (CLIA) -approved lab is required prior to making any changes to their health care. All confirmation testing is completed through Ambry and costs are covered with research funds to eliminate real or perceived cost barriers to confirming results. Testing kits are mailed to the participant’s home with instructions for how to provide a sample. Once results are available, confirmation test results are provided according to the original randomization arm (e.g. by GC or uploaded to the digital-ROR intervention). Once results are confirmed, the clinical test report and summary of the return of actionable results are documented in the EHR.

Multiple follow-up contact attempts are made to participants who do not complete confirmation testing. Three phone call attempts are made by the research team, and a confirmation testing follow-up chat (Chat 3) is sent to all participants who do not complete confirmation testing. Initial chat notification is sent 7 days after confirmation testing kit is ordered, followed by 3 reminders. Participants who have not completed confirmation testing will receive a Confirmation Testing Closing Letter (Supplemental Fig. 4) to reiterate the importance of confirmation testing and invite them to reconnect with the program to complete this step.

#### Clinical genetics referral

All participants with clinically confirmed results are recommended to the appropriate clinical genetics program for expert clinical recommendations and to facilitate appropriate clinical care based on their genetic test result. The participant’s contact information is provided to these clinical teams to schedule the clinical visit. The number of contacts for scheduling is based on the standard clinical procedures for each of the clinical programs. For participants who are no longer local, information about the local clinical genetics program is provided.

Those who have not completed clinical genetics follow-up are provided a Clinical Follow-up Closing Letter, reiterating the importance of this clinical appointment and inviting them to reconnect with the program for assistance in getting a referral (Supplemental Fig. 4).

### Outcomes

#### Conceptual model

Understanding the outcomes of receiving genetic results will be critical to realizing the risks and benefits of returning actionable genetic research results. The effectiveness of returning genetic information is contingent upon successful behavior modification (e.g. performance of risk-reductive interventions and communication to relatives). Thus, our studies evaluating delivery innovations in genetic medicine have been informed by our conceptual model grounded in The Self-Regulation Theory of Health Behavior (SRTHB) [80–87]. SRTHB has been utilized in intervention-based research involving the study of health threats and behaviors [88–90]. It proposes that the reaction to and use of health information is the product of an individual’s understanding and the biopsychosocial impact of the risk and the health behavior. It emphasizes “common-sense” representations rather than medical or scientific definitions, and incorporates individual, biological, cognitive, emotional, familial and cultural experiences that might contribute to individual variability in outcomes [82, 91, 92]. The literature and our data support the hypothesis that cognitive (knowledge and perceptions), psychological (e.g. distress, uncertainty) and behavioral (risk reducing behaviors and communication to relatives) outcomes will be moderated by test result [93–96], cancer and cardiovascular history [94, 97–99], sociodemographic factors, e.g. education, race/ethnicity [94, 99–101], and cognitive and emotional factors, e.g. health literacy [102–104] (Supplemental Fig. 5).

Even after health-related interventions have proven effectiveness, many fail to translate into clinical settings [105, 106]. Thus, there is increasing recognition of the importance of evaluating and addressing barriers to implementation across diverse health care settings. The Consolidated Framework for Implementation Research (CFIR) provides an overarching theoretical framework to evaluate barriers, enhancements and adaptations to increase successful implementation of effective interventions and delivery adaptations [107]. We have included constructs from each of the 5 major CFIR domains as they relate to our return of results approach and eHealth delivery alternatives (Supplemental Fig. 5), which will be evaluated in our multi-stakeholder mixed-methods process evaluation. Evaluation of CFIR components is expected to identify patient, provider and system barriers and enablers of our return of results approach and increase future implementation in other research and health care settings [108–110]. 

#### Uptake of results and patient reported outcomes (aims 1 and 2)

Theoretically informed patient-reported cognitive, affective and behavioral outcomes of genetic services are collected at baseline (T0), 2–7 days post-disclosure (T1) and at 6 months (T2).

1. Knowledge of genetic disease (primary non-inferiority outcome) is evaluated at all time points (T0-T2) using an adapted version of the KnowGene Scale, a 16-item scale administered to patients after genetic testing and/or genetic counseling to measure their understanding of the health implications of genetic test results [111]. It includes health implications to oneself as well as relatives. This measure covers penetrance, actionability, limitations of current technology, and monogenic inheritance patterns. Items 3, 5, 13 and 15 were excluded as they are not relevant to return of actionable results. Additionally, 4 items evaluating understanding of the difference between research and clinical testing and utilized in the RESPECT2 study were added to create a final scale of 16 items. This adapted scale has had good internal consistency in our pilot and related studies (Cronbach’s α = 0.63–0.83) [79, 112]. Perceived risk of cancer and cardiovascular disease (T0-T2) is evaluated with a numerical scale (risk 0-100%) and an 5-point verbal scale (much lower than average risk to much higher than average risk) as utilized in our related studies. The 8-item Attitudes Toward Genetic Testing scale is assessed at baseline to understand perceptions of genetic testing and to assess informed choice in combination with genetic knowledge and decisions to proceed with testing, as previously utilized in the multi-dimensional measure of informed choice and our related research (Cronbach’s α = 0.73) [113–115].
2. *Reactions to genetic information* are assessed with multiple instruments that evaluate psychological distress, adjustment and satisfaction. (a) Disease-specific distress (primary non-inferiority outcome) is measured at all timepoints (T0-T2) using the 8-item version of the Revised Impact of Events Scale (RIES) [94, 116–121], evaluating intrusive and avoidant thoughts regarding cancer and cardiovascular disease separately. The 8 item scale has strong internal consistency in related studies and our pilot study (Cronbach’s α = 0.70–0.92) [122, 123]. (b) *General anxiety and depression* are assessed by the respective 4-item short Patient Reported Outcomes Measurement Information System (PROMIS) scales (Cronbach’s α = 0.88 and 0.86) [79, 124]. (c) *Satisfaction with genetic services* (T1 only) will be assessed with a 14-item satisfaction with communication scale, and adapted for telehealth and digital disclosure, evaluating participants’ experience with high internal consistency in our related studies (Cronbach’s α = 0.73–0.85) [112, 114, 125] (d) *Multidimensional responses to genetic testing*, including positive responses and uncertainty, are assessed at T1 and T2 using the 20 items from the Multi-dimensional Impact of Cancer Risk Assessment Questionnaire (MICRA) with strong internal consistency in our pilot study by subscales (Cronbach’s α = 0.77–0.85) [79, 126]. (e) Decisional regret (T1, T2) is assessed using with the 5-item validated Decision Regret Scale used in genetic studies [127, 128]. This scale has had strong internal consistency in our pilot study (Cronbach’s α = 0.86) [79].
3. *Use of genetic information* includes uptake of receipt of actionable research results and other health and communication behaviors. (a) *Uptake of research results (secondary non-inferiority outcome)* will be assessed as completion of a remote telehealth disclosure session with a GC (either arm) or as viewing results in digital-ROR (digital arm). Other secondary uptake behaviors include uptake of supplemental digital education (Aim 2a), assessed as completion of pre-disclosure digital education or Chat 1, actively opting-out of receipt of research results after the Step 1 or Step 2 Letter (Aim 2b), completion of confirmation testing, and completion of clinical genetics follow-up, either by documentation in the EHR or self-report by those who are no longer in the local area. (b) Risk reducing behavioral intention (T1 only) and performance (T2) will be assessed with a close-ended and open –ended items given a lack of established measures and the wide range of potential medical management options depending on the individual gene. General health status and risk modifying behaviors (e.g. diet, exercise, tobacco and alcohol use) will be assessed at baseline and at T2 with items utilized in BRFSS and related studies [73, 125, 129]. Additionally, gene-specific medical management or risk reducing screening and interventions will be abstracted from medical records. (c) Communication of genetic test results includes communication intent (T1 only) and performance (T2) assessed using a 3-item measure of intent and communication to assess intent to share genetic test results with health care providers, family members and other third parties utilized in related research [85–87].

#### Moderators of uptake and patient reported outcomes (aim 3a)

Moderators are collected at baseline and include sociodemographics, health literacy, comfort with technology and general health status. Sociodemographic data includes race/ethnicity, education, marital status, gender, age, zip code, health insurance status, usual source of care status, employment status, financial wellness [130] and household income. Test result, including cancer versus cardiovascular/other actionable result and the specific gene are recorded. General health status [129] and personal and family history of cancer and cardiovascular disease are included in the baseline survey. Health literacy is assessed at baseline (T0) with 3 items validated to detect inadequate health literacy in clinical medical populations [131]. Comfort with technology is assessed with selected items from the NCI Health Information National Trends Survey (HINTS), including internet and social media use electronic medical record use and perceptions of privacy (7 items) [132].

Additionally, individual use of digital interventions are recorded throughout the study to evaluate the impact of intervention usage (e.g. dosage) on outcomes and who benefit from supplemental digital education or digital-ROR.

#### Multi-stakeholder mixed-methods process evaluation (aim 3b)

CFIR-informed constructs have been selected to evaluate factors related to uptake of our digital delivery model, and implementation facilitators, barriers and adaptations. We will collect additional quantitative and qualitative data from multiple key informants participating and not participating in the study. Proposed key informants include biobank participants, GCs, medical providers, including genetic specialists and PCPs, and research and clinical support staff. Other proposed key informants not participating in the study include other research teams, health system administrations, information technology staff, and insurers. Sources for our mixed methods process evaluation will include participant surveys, research meeting notes and records and key informant interviews as outlined in Supplemental Table 1.

### Data analysis plan

#### Primary non-inferiority analyses

We hypothesize that disclosure of actionable results by digital-ROR, with access to a GC, can provide non-inferior short-term and longitudinal cognitive, affective, and behavioral outcomes, and lower costs. Primary effectiveness outcomes include: post-disclosure knowledge and cancer or cardio disease-specific distress. We will test whether the receipt of results differ between arms as a secondary analysis. Receipt of results is considered a health behavior. For the 2 primary analyses, we will apply non-inferiority tests to examine if access to digital-ROR is non-inferior to GC disclosure. We will conclude non-inferiority if the upper bound of the randomization effect 97.5% one-sided confidence interval [CI] for cross-sectional T1 responses do not cross a -0.45 standardized difference threshold (i.e., margin) for knowledge and a 0.2 standardized threshold (i.e., margin) for disease-specific distress in the direction that would suggest that digital-ROR is worse. A standardized difference is the difference after variables are scaled to have standard deviation (SD) one with respect to baseline SD. We based the margins on what might be considered clinically relevant effects given our pilot data. We will use intention-to-treat approaches, comparing randomized arms, with confirmation by per-protocol analyses (i.e. restricting sample to those who properly used the method of disclosure as assigned). We will only declare non-inferiority if both intention-to-treat and per-protocol analyses are consistent, as recommended [133, 134]. For the secondary outcome receipt of results, we will examine non-inferiority of proportions. We will declare non-inferiority if the upper bound of the randomization effect 95% one-sided confidence interval for receipt of results is < 10% in the direction that favors usual care. Since all participants will have the option to speak with a GC, we will conduct secondary as-treated analyses (i.e. based on whether genetic counseling was actually received in both arms).

In secondary analyses, we will use T-tests and Fisher’s exact tests for superiority testing. We will assess balance of potential confounders (e.g. age, race, study site) between arms using T-tests or Fisher’s exact tests. We will use multiple linear regressions (continuous outcomes) and logistic regressions (presence of a behavior such as receipt of results or uptake of confirmation testing) to examine effects after adjusting for potential confounders inadequately balanced. For longitudinal analyses that include all three time points (T0, T1, and T2), we will estimate regressions by Generalized Estimating Equations (GEEs); we will include main and interaction terms between arm and time [135]. 

Intervention costs will be estimated for each study arm, and mean costs per participant will be reported. We will perform a benefit-cost analysis comparing the two study arms, where net benefit (or cost) of the intervention is estimated as the difference in mean downstream costs (i.e., savings in health care expenditures attributable to the intervention) minus the difference in mean intervention costs. In sensitivity analysis, we will vary both intervention cost items and the costs of downstream health care services over reasonable ranges and examine the impact on results. The time horizon of the benefit-cost analysis will be limited to the 12-month follow-period. We will not project longer-term outcomes, nor will we estimate the potential economic impact on family members of genetic test results in study participants, as these are beyond the scope of this study.

#### Sample size justification for aim 1

We chose *n* = 255/arm so we could conclude non-inferiority of digital-ROR if randomization effects comparing the digital arm to GC arms at T1 do not show clinically relevant worsening in the web arm. We chose a margin of -0.45 standard deviation units for knowledge and 0.20 for distress since they are considered modest changes in behavioral research, and they are consistent with our pilot data. In preliminary data from our pilot study, the baseline knowledge standard deviation was 2.86 points. A -0.45 standard deviation difference * (times) 2.86 = -1.29 points. A 1.29 point worsening in the knowledge scale would not be considered a meaningful difference. For disease specific distress, the baseline standard deviation was 9.31 points. A 0.20 standard deviation difference * (times) 9.31 = 1.86 points. Again, a 1.85 unit worsening in the distress scale would not indicate a clinically relevant change.

Table 4 demonstrates potential allowable non-inferiority differences. We chose 2.5% Type I error by applying a Bonferroni correction to a 5% Type I error rate with 2 comparisons (5%/2 = 2.5%). We calculated power using simulations in R with confirmation using PASS 11 software. For our secondary outcome of receipt of results, we will declare digital-ROR effect non-inferior if the upper bound of the 95% one-sided confidence interval effect is less than 11% in a direction that would favor usual care. This has an approximate 5% non-inferiority Type I error rate and > 80% power over a range of plausible differences given our preliminary data (e.g. >55% promising rate seen in preliminary data versus < 44% discouraging rate, *n* = 255/arm). We assumed a binomial pooled standard error estimator. We do not correct for multiple testing of secondary outcomes.

**Table 4:** Estimated non-inferiority power, 2.5% Type I error (1-sided), number = 255/group (510 total)

Variable	Biobank Wave 2 Pilot Study	Non-inferiority difference allowed	Non-Inferiority Type 1 Error Rate and Power
Knowledge	T1 Mean 13.20, SD = 2.59 GC arm T1 Mean 12.50, SD = 2.12 Web arm Web minus GC effect = -0.7 Baseline standard deviation = 2.86	-0.45 SD units * 2.86 base SD = -1.29 point margin	2.5% Type I error rate > 80% power with − 0.7 pilot data effect > 99% power with 0 point effect
Disease-specific Distress	T1 Mean 6.22, SD = 5.72 GC arm T1 Mean 6.00, SD = 6.42 Web arm Web minus GC effect: -0.22 Baseline SD = 9.31	0.20 SD units * 9.31 base SD = 1.9 point margin	2.5% Type I error rate > 95% power with − 0.22 pilot data effect > 90% power with 0 point effect.

#### Missing data

To account for missing data, we will use multiple imputation by chained equations methods of Raghunathan et al. for primary analyses [136]. In sensitivity analyses, we will perform complete case analyses with the anticipation that doing so will confirm the conclusion.

#### Interim analyses

We conducted an interim analysis after 100 people per arm had been enrolled and completed the T1 post-disclosure survey (200 total who have completed the T1 survey). We constructed the interim analysis to protect the Type I error rate. We did not pause the study based on the interim analysis.

#### Aim 2 analyses

Estimation of the proportion opting out will constitute our primary Aim 2 analysis and estimation of the proportion with uptake of supplemental digital education will constitute our secondary Aim 2 analysis. We will also estimate 95% confidence intervals with the proportions. For our primary Aim 2 analysis, we will test whether the proportion opting out is 5% using a one sample binomial exact test. We will primarily assess the proportions opting out with respect to those who are evaluable (i.e., alive and who do not have returned mail). We will subsequently assess proportions among those evaluable and who have an actionable result (secondary analysis). We will also compare opt-out between cases and controls.

For our secondary Aim 2 analysis, uptake of digital education will be classified as (1) accessing digital education, and (2) completing digital education. Secondary outcomes of education uptake will include (3) the platform used (web or chatbot), (4) # of visits to digital education, (5) total time, and (6) accessing videos. We will describe secondary outcomes using means and standard deviations (e.g., time and # of visits) or proportions (e.g., accessing individual videos) as appropriate. To evaluate the impact of completing education on opting out, we will compare opt-out rates between those who do and those who do not access and complete digital education using Fisher’s Exact Tests (Aim 2c). In secondary analyses, we will use generalized linear models with appropriate link and family functions for Aim 2 endpoints (e.g., Poisson regressions for # of visits, linear regressions for baseline patient reported outcomes). We will enter confounders into the models. We will examine models separately for those who did and those who did not receive letter 1 or letter 2.

#### Sample size justification for aim 2

We anticipate an approximate 5% opt-out rate based on our pilot data. With at least 1250 participants (and up to 2500 participants), we will be able to test whether the rate is < 5% (null hypothesis) versus > 7.5% (alternative hypothesis) with 95% power and a 2.2% Type I error rate (one-sided), and the use of a one-sample binomial test.

#### Aim 3 analyses

We hypothesize that moderators will identify those who benefit more and less from the eHealth interventions and can inform policies for return of results. For moderation (i.e., effect modifiers), we will include in regressions described in Aim 1 an indicator variable for randomization arm, panel time indicators (for longitudinal models examining all three time points), the potential moderator variable, and all two-way and relevant three-way interactions among the arm/time/moderator variables [135]. To maintain power, we will examine non-time moderators separately.

Qualitative data will be analyzed using an iterative, deductive content analysis approach and CFIR as the coding framework [107, 110, 137]. The qualitative data will include: (a) participant surveys and key informant interviews; (b) notes and debriefing from our research team meetings. Guided by consensual qualitative methods [138, 139], two independent coders will review notes and assign responses to CFIR constructs. Using a convergent mixed-methods approach [140], we will merge quantitative and qualitative data, organized by the 5 CFIR domains to evaluate which constructs were associated with, or more prevalent among: (a) patients who used the digital interventions exclusively versus those who used digital interventions and requested a GC versus those who requested to speak with a GC and declined use of digital interventions, (b) participants who used chatbot options versus web-based options, (c) participants who had confirmation testing and clinical medical follow-up versus those who did not, and (d) those who shared results with multiple relatives versus those who did not, providing an opportunity to comprehensively evaluate facilitators and barriers to implementation of digital alternatives for return of actionable genetic research results in large biobanks and research cohorts. We will use descriptive statistics, correlations, and generalized linear models, assuming appropriate link and family functions, to examine quantitative variables associated with barriers and uptake. We will estimate longitudinal models by Generalized Estimating Equations.

## Results

This study is ongoing and is expected to complete Aim 1 in 2026.

## Discussion

We hypothesize that digital return of genetic research results will result in equal or improved short-term and longitudinal participants cognitive, affective and behavioral outcomes, and lower costs, providing a scalable model for returning actionable results to research participants in large biobanks. Results from this trial will be presented at national conferences and published in peer-reviewed journals. This digital alternative has the potential to reduce the overall costs of returning genetic research results, although we acknowledge that there are multiple steps in the return of results process and other related costs to be considered. We expect to have data to describe the overall costs of our approach to further inform best approaches and anticipated costs of return of genetic research results in large scale biobanks where return of results is not a primary goal of the research. Furthermore, we expect to evaluate the uptake of pre-disclosure education, its impact on opting out of receiving actionable genetic results and participant outcomes with return of results, and the barriers to future adaptation and implementation of digital interventions for return of genetic research results.

### Limitations and potential challenges

We acknowledge several potential challenges. Some participants may not have web or digital access, although we expect this will not be a barrier for most participants. Our interventions are mobile ready and those randomized to the intervention arm will still have the opportunity to receive results with a GC by phone. It is possible that interest in digital options may be lower than expected in a diverse clinical population. While our pilot data and related studies suggest high interest in digital alternatives [141–144], even if there are frequent requests to speak with a GC in the intervention arm, it will provide critical data regarding participant preferences and required resources and costs. And, it may still reduce GC costs as those who request a call with a GC after receipt of results may have shorter and more targeted discussions. While not anticipated, it is possible that we reach fewer participants than projected, that opt-out rates could be higher or that we could identify fewer actionable mutations than predicted.

### Potential for impact and implications

The RESPECT3 study is expected to provide critical empiric data on the effectiveness of using digital alternatives for pre-disclosure education and return of genetic research results in a representative clinical population of research participants who enrolled in an institutional biobank where return of results was not the focus of the study. Equally important, our theoretically-informed process evaluation data is expected to inform modifications to identify barriers and facilitators to broader implementation.

## Supplementary Information

Supplementary Material 1.

## Data Availability

Data is available upon request.
