# Supplementary material for: A randomized study of digital versus genetic counselor return of actionable genetic research results to biobank participants (RESPECT3 study)
Source: BMC Med Ethics. 2026 Mar 31;27:122. doi: 10.1186/s12910-026-01439-x (PMC13340395; doi:10.1186/s12910-026-01439-x)
Supplement: Supplementary file 1 — Supplementary Material 1. [file 12910_2026_1439_MOESM1_ESM.docx]

**Supplemental Figures**

**Supplemental Figure 1** Step 1 Letter and Flyer


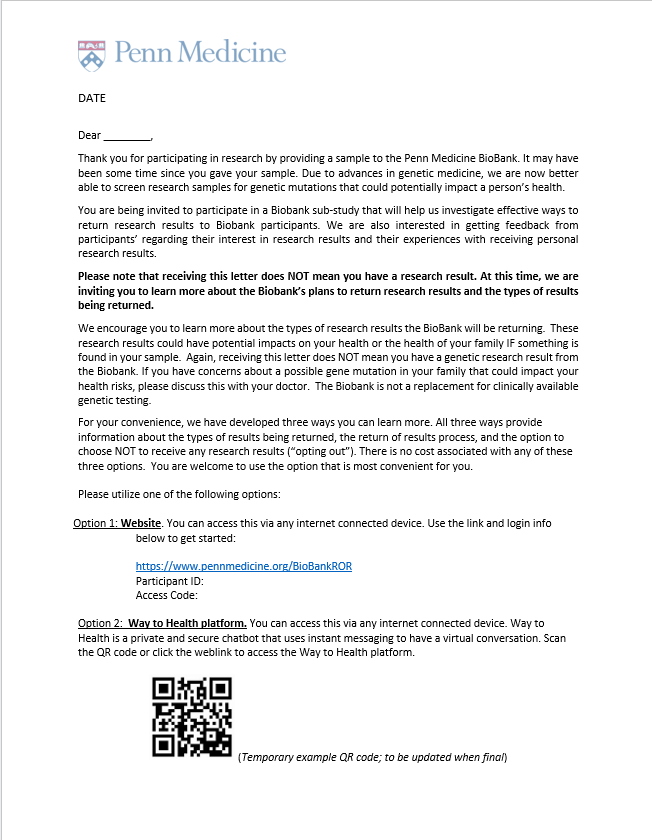


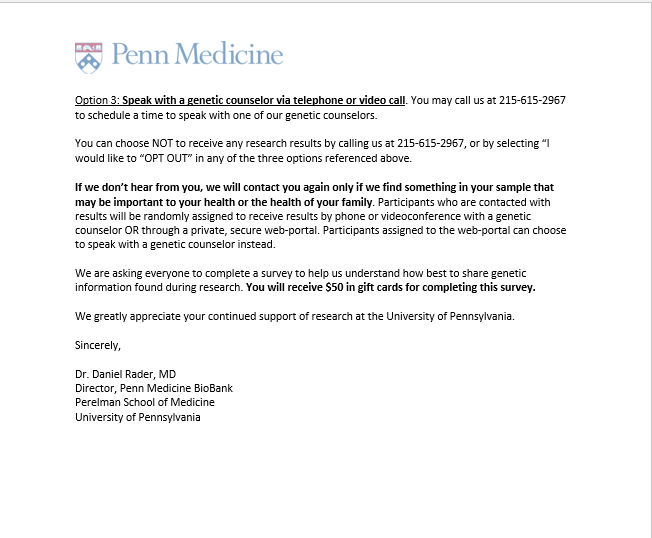


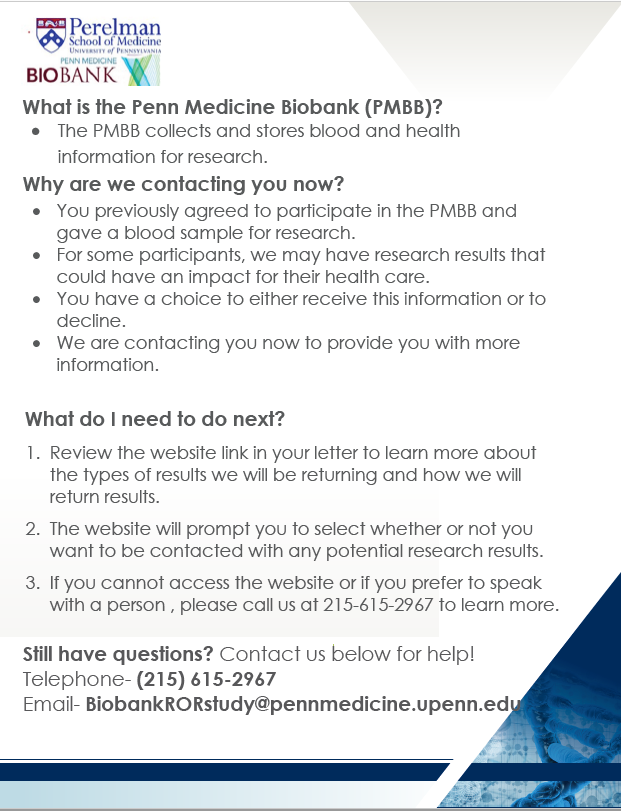


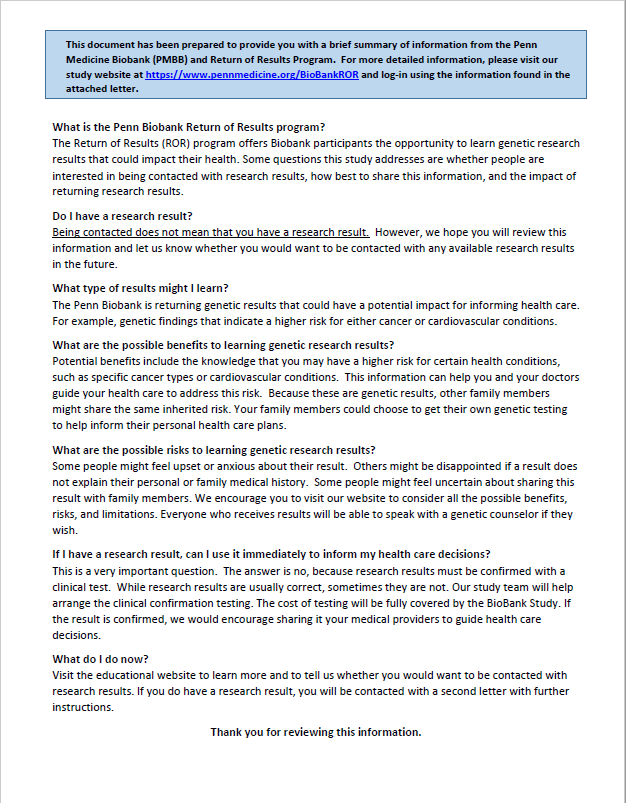


**Supplemental Figure 2a** Step 2 Letter (Arm 1)


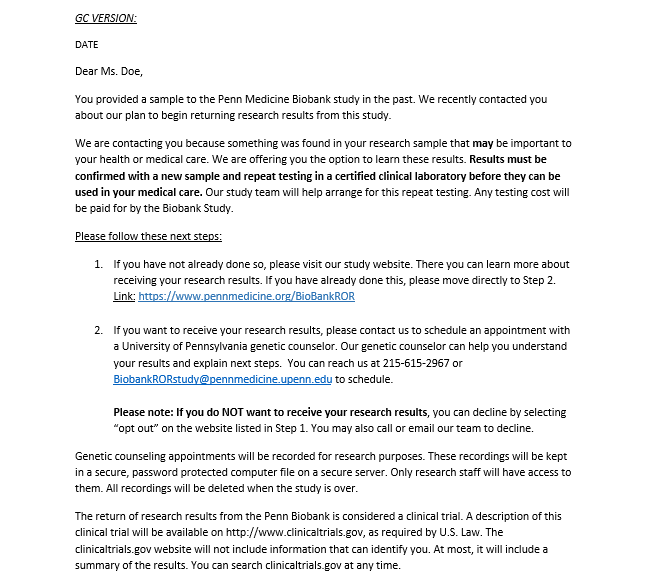


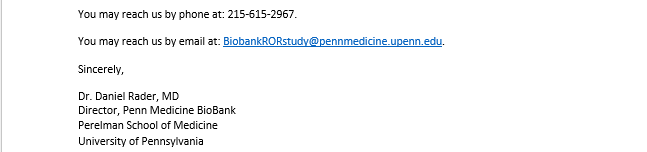


**Supplemental Figure 2b** Step 2 Letter (Arm 2)


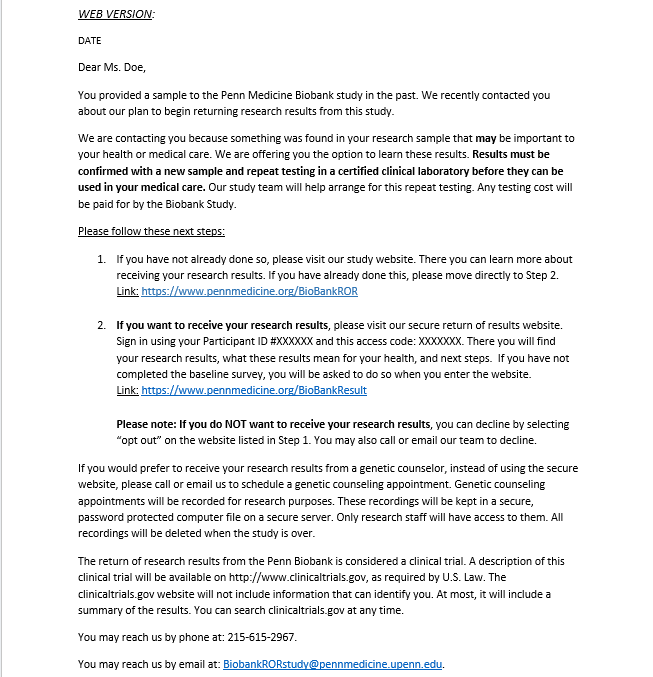


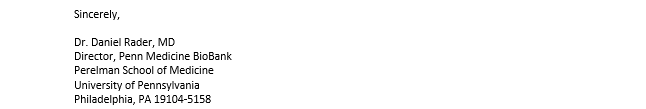


**Supplemental Figure 3:** Genetic research result report


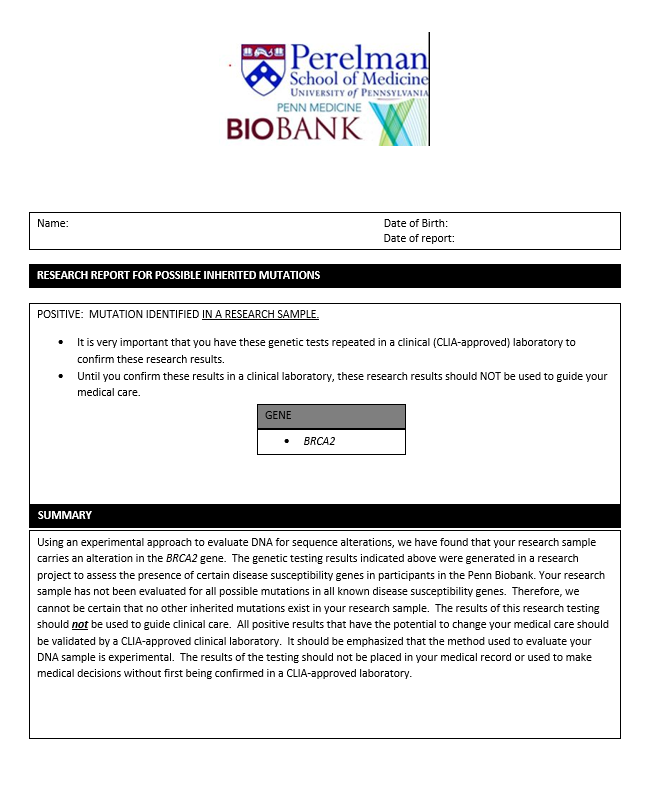


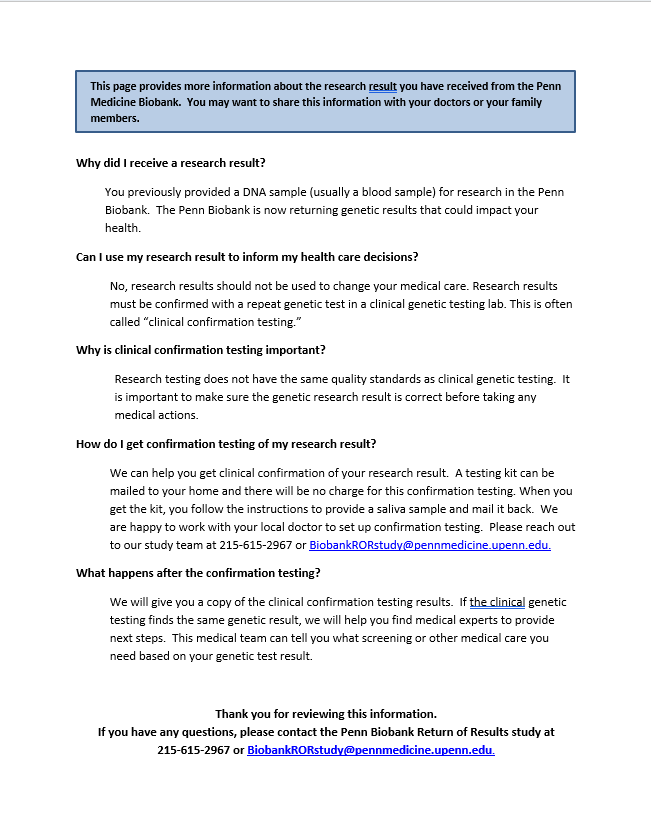


**Supplemental Figure 4** Closing letters

**Supplemental Figure 4a** Closing letter for step 2 letter


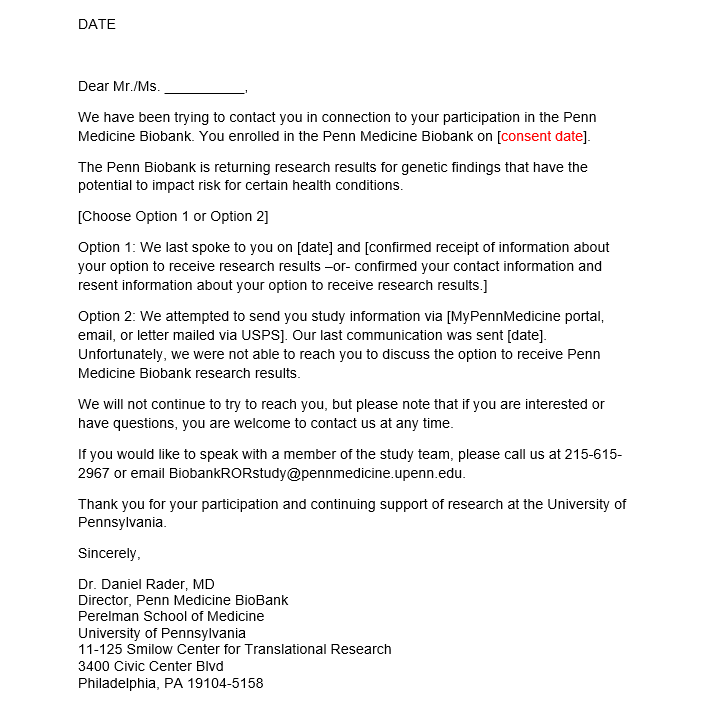


**Supplemental Figure 4b** Closing letter for clinical confirmation testing:


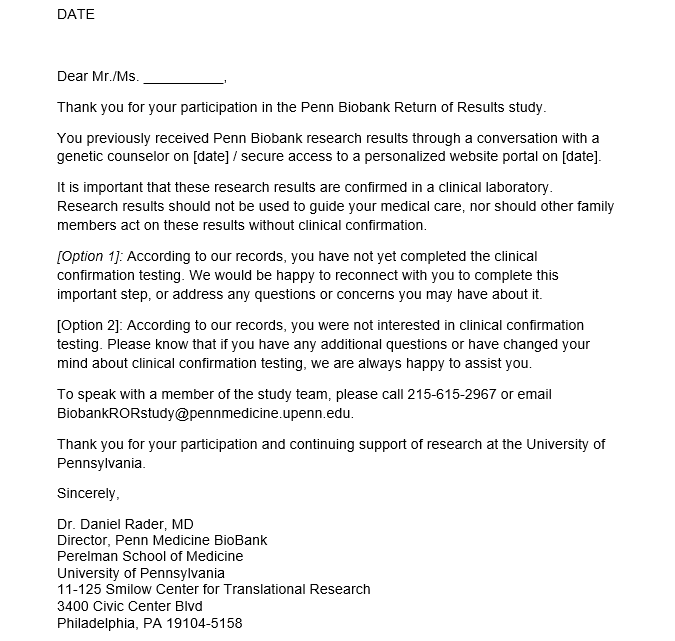


**Supplemental Figure 4c** Closing letter for clinical follow-up


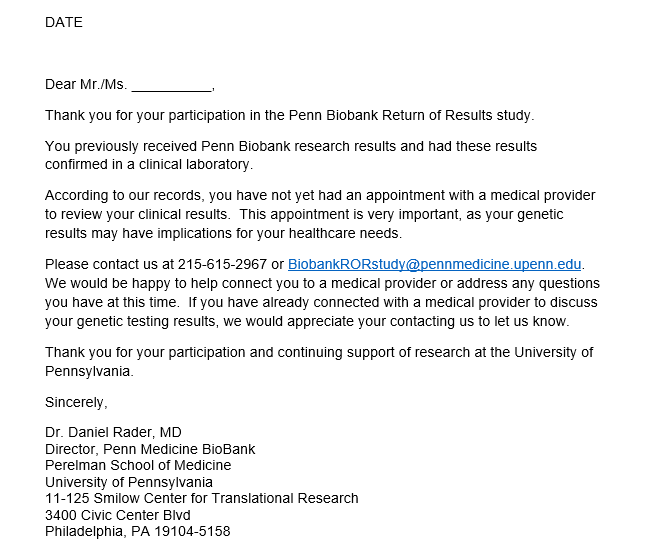


**Supplemental Figure 5** Conceptual Model to evaluate innovations to delivery of genetic services: guided by the Self-Regulation Theory of Health Behavior (SRTHB) and Consolidated Framework for Implementation Research (CFIR)

**Delivery innovation: Digital alternatives to pre-disclosure education and return of results**

Notification of:

a) Access to digital education b) Choice to opt-out of receipt of actionable genetic research results **(Aim 2)**

**Effectiveness (Aim 1):**

a. Short-term and longitudinal SRTHB informed patient outcomes and cost

-Understanding (Knowledge and perceptions)

-Reaction to ROR (e.g. distress, uncertainty)

-Use of ROR (uptake of results, screening/preventive measures, and communication to at-risk relatives)

b. Genetic counselor time and costs

:

**Moderators of uptake of ROR and SRTHB Patient Reported Outcomes (Aim 3a)**

Sociodemographics (i.e. age, race/ethnicity, education, gender, income, employment status), personal and family history, health literacy, comfort with technology, genetic research result

**CFIR Attributes of successful Implementation (Aim 3b)**

-Intervention characteristics (relative advantage, adaptability, complexity, cost)

-Outer setting (research team providers, health system and insurers)

-Inner Setting (structural characteristics, climate implementation, readiness for implementation)

-Characteristics of individuals (type of research cohort/health system, comfort with technology literacy, and comfort with technology)

-Process (planning, champions, opinion leaders, reflecting)

**Supplemental Table 1** Consolidated Framework for Implementation Research (CFIR) constructs, measures, sample items and sources.

| **Measures** | **Sample Items** | **Participant**  **Survey** | **Research meeting records** | **Key Informant Interviews*** |
| --- | --- | --- | --- | --- |
| **Intervention Characteristics** | | | | |
| Relative advantage | Perception that digital delivery alternatives provides an advantage over usual care (pre-disclosure and disclosure counseling with a GC) | X |  | X |
| Adaptability | Perception that digital delivery alternatives could be modified to meet research program, provider or patient needs | X |  | X |
| Complexity | Perception that digital delivery alternatives are too complex | X | X | X |
| Cost | Perception that digital delivery alternatives are too costly |  |  | X |
| **Outer setting** | | | | |
| Research team, health system, provider, insurer needs and resources | Awareness of value of returning actionable genetic results and utilizing digital service alternatives, disruption to research program, health system, clinical providers or insurers |  |  | X |
| **Inner Setting** | | | | |
| Structural characteristics | EHR, support staff characteristics and needs, practice structure (medical providers* and GCs) |  | X | X |
| Climate for implementation | Compatibility with practice/system, relative priority, quality metrics |  | X | X |
| Readiness for Implementation | Available staff resources, experience with digital services |  | X | X |
| **Characteristics of Individuals** | | | | |
| Health system, research team and clinical team background | Type of research cohort and health system, exposure to digital tools |  |  | x |
| Participant background | Age, race, ethnicity, education, insurance | X |  |  |
| Participant attitude toward genetic testing | Attitudes about genetic testing scale (8 items) | X |  |  |
| Participant comfort with technology | internet use (8 items), electronic medical record use and perceptions of privacy (14 items) | X |  |  |
| Participant’s baseline knowledge, health literacy and affect | See patient reported outcomes | X |  |  |
| **Process** | | | | |
| Planning | Workflows for support staff and medical providers, quality of materials describing procedures for patients and medical provider supports |  | X | X |
| Opinion leaders | Which research/clinical staff, health system and providers are most influential? How do they influence others and what could have helped |  |  | X |
| Champions | Who in the program/health system helps ensure all steps were completed? What did they do to make digital alternatives successful? |  | X | X |
| Reflecting and evaluation | What procedures are working (recontact, scheduling, digital tool access, chat-bot features, medical follow-up and confirmation testing) and not? What can we change to make the process easier? etc. |  | X** | X** |
| Medical providers include genetic specialists in cancer, cardiovascular and medical genetics, GCs (genetic counselors) and PCPs ( primary care providers) ; ***KEY INFORMANTS** will include selected participants (n=15-30); genetic specialists (7-12), PCPs (15-30) genetic counselors not participating in the study (15-30), other research teams, health systems administrators, informatics specialists and insurers (5-8 each); ** includes regular personnel and team debriefing about what is working and not working about the return of results process or digital interventions | | | | |
